# Crafting Pyrolysis-Free M–N–C Catalysts

**DOI:** 10.1021/prechem.3c00026

**Published:** 2023-04-14

**Authors:** Chang-Xin Zhao, Qiang Zhang

**Affiliations:** Beijing Key Laboratory of Green Chemical Reaction Engineering and Technology, Department of Chemical Engineering, 12442 Tsinghua University, Beijing 100084, China

**Keywords:** M−N−C catalysts, electrocatalysis, porphyrin, phthalocyanine, organic frameworks

## Abstract

M–N–C catalysts, with their transition metal atoms coordinated to nitrogen/carbon atoms as active sites, are gaining attention for their versatile heterogeneous electrocatalysis. Fabrication of these catalysts can be achieved through either bottom-up chemical synthesis or top-down pyrolysis procedures, where the former offers a well-defined structure and precise synthesis feasibility. This Perspective provides an overview of the history of the technical route dispute between pyrolysis and pyrolysis-free M–N–C catalysts, along with their respective advantages and disadvantages. Additionally, we emphasize the advantages of pyrolysis-free M–N–C catalysts, exemplified by several studies focused on precisely modulating the structure to regulate the activity, as well as the efforts of effectively integrating the active sites. Finally, we discuss the challenges and opportunities of pyrolysis-free M–N–C catalysts, with the aim of maximizing their inherent strengths of precise structure and promoting their industrial applications.

## Graphical abstract

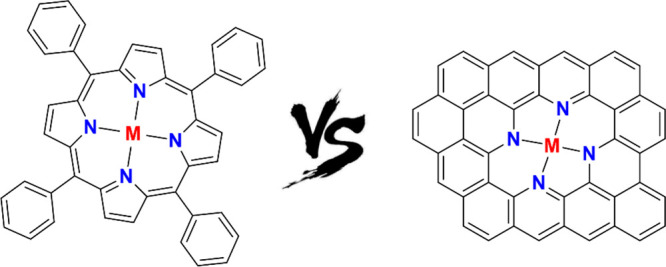

## 1 Introduction

Catalysis is a phenomenon that is ubiquitous in nature and has been an integral part of the chemical industry throughout its history.^1,2^ For catalytic investigation, the two fundamental goals remain constant. First, it is essential to be able to construct active sites with a well-defined structure in a controllable and precise manner. Such a capability is paramount for bottom-up molecular design, intrinsic activity regulation, and structure–performance relationship investigation.^3^ The second is the pursuit of performance, including activity, selectivity, stability, etc.^4,5^ However, the above two goals are somewhat contradictory, or at least hard to balance for several catalytic reactions or catalyst systems. M–N–C catalysts, a class of heterogeneous electrocatalyst with metal atoms coordinated with N/C atoms serving as the active sites,^6–10^ face such a dilemma. Specifically, M–N–C catalysts can be fabricated via a bottom-up chemical synthesis or a top-down pyrolysis procedure. On one hand, the pyrolysis-free chemical synthesis route guarantees the precise construction of active sites of M–N–C catalysts, but the resulting catalyst suffers from conductivity and stability concerns.^11^ On the other hand, the top-down pyrolysis procedure ensures satisfactory electrocatalytic performance, while the pyrolysis process also sacrifices the capability of precisely constructing the active sites as the pyrolysis process increases the uncertainty of their structure.^12^

This technical route dispute on M–N–C catalysts’ preparation has lasted for decades since the 1970s. Yet in the recent decade, the pyrolysis route seems to have the upper hand. Tremendous efforts have been devoted to the pyrolysis route to fabricate M–N–C catalysts, where diverse types of precursors have been selected, such as polymers,^13,14^ metal–organic frameworks,^15–18^ covalent organic frameworks,^19^ biomass,^20^ etc. Recently, research in this field has encountered an unprecedented bottleneck: while the pyrolysis synthesis technique has experienced rapid development, precisely fabricating active sites on the basis of reaction mechanisms has remained largely stagnant. The failure to create bottom-up molecular design or precise structure construction has impeded further electrocatalytic performance enhancement. For instance, in 2017, an Fe–N–C catalyst prepared via pyrolysis synthesis achieved an electrocatalytic oxygen reduction (ORR) activity of *E*_1/2_ = 0.90 V,^21^ yet little improvement has been achieved in the following six years until now. More attention should be paid to pyrolysis-free synthesis routes, taking useful inspiration from this research paradigm to guide bottom-up molecular design and precise active site construction.

Through this Perspective, our goal is to comprehend the reasons and methods behind pyrolysis-free synthesis and its contribution to the advancement of M–N–C catalysts from both theoretical and practical perspectives. To that end, we conduct a concise historical review of the pyrolysis/pyrolysis-free technical route dispute over the past few decades. We further analyze the advantages and disadvantages of pyrolysis-free synthesis, along with inspiring examples on how to maximize the advantages and minimize the disadvantages. Additionally, we provide critical comments to compare the pyrolysis and pyrolysis-free routes in order to gain an unbiased view on the further development of advanced M–N–C catalysts, reconciling the contradictions between precise structure and practical performance. By joining and focusing on multidisciplinary knowledge, the era for creating pyrolysis-free M–N–C catalysts as the next-generation electrocatalysts is coming.

## 2 Historical Technical Route Dispute

Before historical narration, a brief introduction to the pyrolysis-free M–N–C catalysts is provided herein, especially for the basic chemistry of metal–macrocycle complexes. Although diverse molecular ligands can be employed to construct the M–N/C coordination structure as the active sites,^22^ the metal–macrocycle is one of the most typical and the most common units for the pyrolysis-free M–N–C catalysts. Typically, the core of the M–N–C catalysts is the center metal atom (M) and coordination atoms in-plane (N and C in most cases) forming coordination structures as the active sites such as the M–N_4_ configured sites.^23^ Several metal–macrocycle complexes possess similar structures to function as the basic units, including metal–porphyrin, metal–corrole, and metal–phthalocyanine.

(1) **Metal–porphyrin:** Porphyrin is a kind of macromolecular heterocyclic compound with four pyrrole subunits interconnected at their α-carbon atoms through methine bridges. Porphyrin is a class of conjugated aromatic compound that satisfies Huckel’s rules. Among four nitrogen atoms in porphyrin, two of them are linked with hydrogen atoms. The deprotonated porphyrin is a dianionic macrocycle and is able to bind diverse kinds of metal ions serving as an excellent chelating ligand due to its proper size to accommodate metal cations. The formed coordination complex called metal–porphyrin demonstrates a four-coordinated structure with a flat square configuration. The axial positions of metal atoms are unoccupied to ensure consequent electrocatalytic functions (Figure 1a).
(2) **Metal–corrole:** Corrole is another kind of heterocyclic compound, belonging to porphyrin families with tight cavities. Corrole shows a similar structure to porphyrin (both are cyclic tetrapyrrolic and conjugated aromatic compounds) but lacks one meso-carbon atom. That is, one pair of adjacent pyrrole units is directly linked through the bonding between their respective α-carbon atoms. Additionally, distinct from porphyrin, the deprotonated corrole is a trianionic macrocycle (as three nitrogen atoms in a corrole molecule are bonded with hydrogen) and possesses a much smaller coordination cavity. Therefore, corrole is a chelating ligand that is more suitable to coordinate high-valent metal cations and form metal–corroles with M–N_4_ configured structures (Figure 1b).
(3) **Metal–phthalocyanine:** Phthalocyanine is another planar macrocyclic conjugated system. It is composed of four isoindole subunits interconnected through nitrogen atoms as the bridge. Phthalocyanine is also a conjugated aromatic compound. The deprotonated phthalocyanine is a dianionic macrocycle and a chelating ligand to coordinate diverse center metal cations. Most of the metal cations can completely enter the deprotonated phthalocyanine cavities to form a planar square M–N_4_ coordination structure, which is named metal–phthalocyanine. The axial positions of center metal atoms are unoccupied, facilitating the interaction between metal atoms and intermediates of electrocatalysis (Figure 1c).

**Figure 1:**
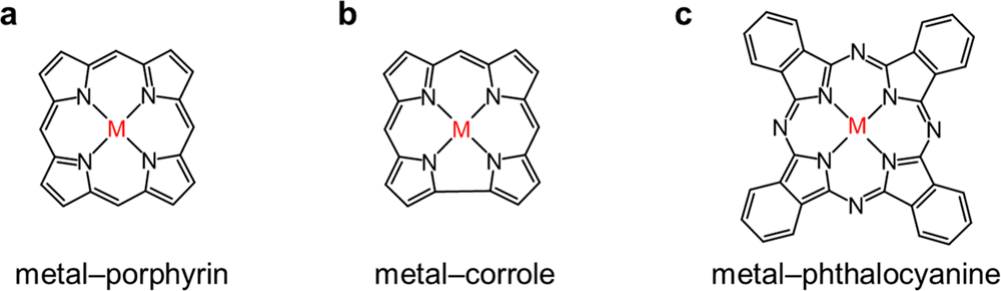
Molecular structures of (a) metal–porphyrin, (b) metal–corrole, and (c) metal–phthalocyanine.

In the past decade, the metal–macrocycle complexes seem to have emerged as the model/reference samples of the pyrolysis-prepared M–N–C catalysts. For example, researchers often use iron–phthalocyanine as the reference when analyzing X-ray absorption spectroscopy results for pyrolysis-prepared Fe–N–C catalysts. However, from a historical perspective, metal–macrocycle complexes are the predecessors of M–N–C catalysts. The first investigation of M–N–C catalysts dates back to 1964, when Jasinski reported the electrocatalytic ORR activity of cobalt–phthalocyanine.^24^ Later on, diverse metal–macrocycles were proposed for different heterogeneous electrocatalysts.^25^ However, the long-term durability of these metal–macrocycles is not satisfactory, partly due to the dissolution of the molecules/central metal atoms into the electrolyte. In 1977, Bagotzky et al. demonstrated that heat treatment of cobalt–porphyrin at 800–900 °C could improve its stability, albeit at the cost of sacrificing the well-defined structure of active sites.^26^ This contribution began the decades-long technical route disputes between pyrolysis and pyrolysis-free M–N–C catalysts. In 2011, Qiao et al. proposed the concept of single-atom catalysis with a demonstration of atomically dispersing platinum atoms onto iron oxide substrate.^27^ Pyrolysis-obtained M–N–C catalysts, which feature atomically dispersed metal atoms on a carbon substrate, are an exemplary example of single-atom catalysts and have been flourishing in the past decade.^28^ To this end, various precursors for pyrolysis have been explored; however, the development of pyrolysis-obtained M–N–C catalysts has stalled in recent years, with minimal progress in terms of electrocatalytic activity. This necessitates the precise construction of active sites in order to achieve a tailored structure and optimized performance. Pyrolysis-free M–N–C catalysts, which are obtained via precise bottom-up chemical synthesis, have well-defined structures and are thus beneficial for both fundamental structure–performance relationship investigations and practical electrocatalytic activity improvement. Consequently, pyrolysis-free M–N–C catalysts have recently been gaining significant attention.

## 3 Precise Construction of Active Sites

In a typical M–N–C catalyst, center metal atoms serve as the actual sites to interact with the reactants/intermediates during electrocatalysis, where the surrounding environment indirectly influences such interactions and consequent electrocatalytic performances.^29^ One of the main advantages of pyrolysis-free M–N–C catalysts compared with those experiencing pyrolysis is the capability of manipulating the active sites, where the electronic structures and the active site configurations can be optimized according to the electrocatalytic mechanisms.

The electronic structure is significantly influential toward the electrocatalytic activity. According to Sabatier’s principle, moderate interaction strength between metal sites and reactants/intermediates corresponds to the maximum electrocatalytic activity,^30,31^ which can be achieved via modulating the electron density of the active sites.^32^ Fortunately, pyrolysis-free M–N–C catalysts afford a supreme platform. That is, specific electron withdrawing/donating substitutions on metal–macrocycle complexes are capable of directionally regulating the electronic structure, adjusting the interaction strength, and therefore enhancing the electrocatalytic activity. For instance, the substitution with electron-withdrawing cyano groups on nickel–phthalocyanine decreases the electron density of active sites, enlarges the energy difference for generating *H_2_O_2_ over *O, and facilitates the 2e^–^ ORR pathway (Figure 2a).^33^ Therefore, nickel­(II) 2,3,9,10,16,17,23,24-octacyano-phthalocyanine functions as an excellent electrocatalyst for H_2_O_2_ electrochemical production. On the contrary, iron/cobalt–corrole/porphyrin starves for higher electron density to achieve its best electrocatalytic activity for 4e^–^ ORR and oxygen evolution (OER).^34–37^ Introducing electron-donating substitutions has been proven to be efficient for activity enhancement. Besides substituting the hydrogen atoms around the macrocycle, introducing the axial groups is also a feasible and even more direct strategy to regulate the electronic structure of the center metal atoms. As an example, the axial imidazole groups in iron–porphyrin systems increase the electron density of iron, facilitate the O–O bond cleavage, and enhance the activity and selectivity for ORR electrocatalysis (Figure 2b).^38^ Such an effect is called the “pushing effect”, which was discovered by Collman et al.^39^ and utilized for fabricating high-performance electrocatalysts by Cao and co-workers.^38^

**Figure 2:**
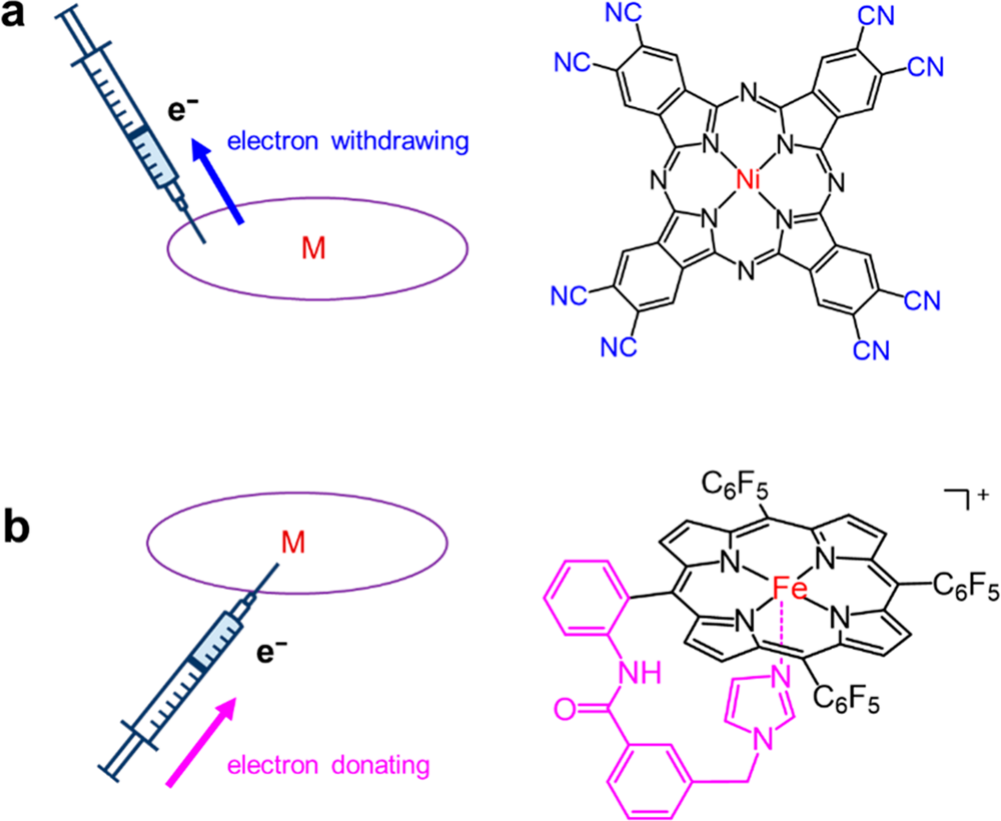
Electronic structure regulation of pyrolysis-free M–N–C catalysts: (a) introducing electron-withdrawing substitutions and (b) “pushing effect” achieved via introducing axial groups.

The configuration of the active sites is another aspect crucial to electrocatalytic activity. Typically, the electrocatalytic reactions undergo sophisticated pathways on the M–N–C sites with multiple intermediates and several proton coupled electron transfer (PCET) steps. Tuning the configuration of the active sites can induce extra interaction with the intermediates besides the pristine metal–intermediate interaction. Such an extra interaction may influence electrocatalysis via multiple mechanisms, regarding the intramolecular proton relay mechanism and bond stretching mechanism. A hangman metal–macrocycle involves a pendant group juxtaposed to the macrocycle and suspends a functional group over the M–N_4_ active sites (Figure 3a).^40^ Specific suspended functional groups, such as carboxyl, afford proton-donating/accepting capabilities and can facilitate the proton transfer process. During the catalytic process, the substrate/intermediate resides in the hangman cleft (between the metal atom and the suspended functional group) and may be activated by PCET, as the proton transfer is delivered from the hanging group and electron transfer is achieved via the metal–macrocycle.^41^ Such a mechanism is named the intramolecular proton relay mechanism. For instance, a hangman metal–macrocycle is effective for ORR, a typical electrochemical process with multiple PCET steps. The proton-donating hanging group can facilitate the protonation of oxygen intermediates, which works in concert with the M–N_4_ sites to activate the O–O bonds, improving the activity and selectivity for 4e^–^ ORR.

**Figure 3:**
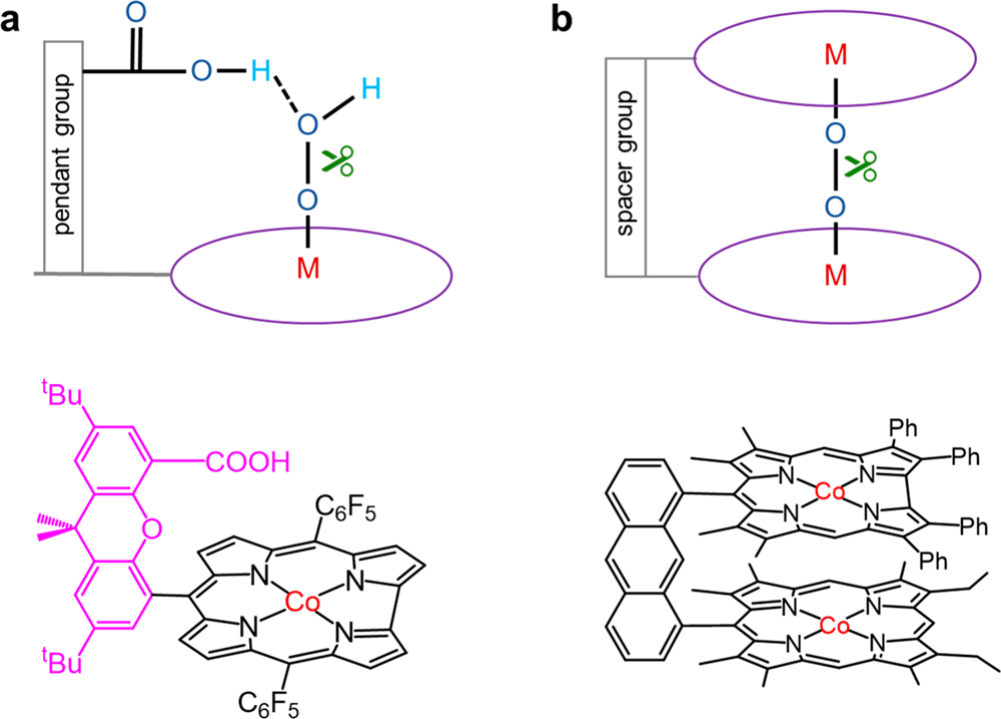
Configuration regulation of the pyrolysis-free M–N–C catalysts, including (a) the hangman metal–macrocycle for the intramolecular proton relay mechanism and (b) the cofacial metal–macrocycle for the bond stretching mechanism.

Another mechanism is called the bond stretching mechanism, which means that specific bonds in the reactants/intermediates are elongated promoting their rupture facilitated by the configuration of active sites. Taking ORR as an example, the key to the 4e^–^ reduction pathway instead of 2e^–^ reduction is the breakage of O–O bonds.^42^ A cofacial metal–macrocycle satisfies the above requirement, where two metal–macrocycles are parallel to each other and are fixed by the spacer group (Figure 3b).^43,44^ The distance between two metal–macrocycles is designed to match the size of oxygen molecule. The oxygen molecule is therefore prone to be adsorbed in the mezzanine of the cofacial metal–macrocycle forming the structure of M1–O–O–M2 (M1 and M2 refer to the metal atoms). That is, the cofacial configuration benefits the trans-Yeager-type adsorption of oxygen which facilitates the stretching and breakage of O–O bonds, instead of the Griffiths- or Pauling-type adsorption which is unfavorable to O–O bond cleavage.^45^

## 4 Precise Integration of Active Sites

As previously noted, the pursuit of pyrolysis-free synthesis is a double-edged sword, offering the benefits of customizable molecule design but also incurring the cost of active site dissolution and subsequent inferior performance. As such, the successful integration of active sites that guarantee a strong affinity with the electrode substrate is of paramount importance. The current efforts can be divided into three categories: π–π interaction, covalent immobilization, and reticular chemistry.

The π–π interaction is a particular type of dispersion force established between unsaturated (poly)­cyclic molecules.^46^ Utilizing π–π interactions is the most straightforward strategy to integrate the metal–macrocycle onto a carbon-based conductive substrate (graphene, carbon nanotubes, etc.), as both of them are inherently conjugated π-systems.^47^ A universal strategy for π–π interaction anchoring is to immerse carbon-based materials into a solution of the metal–macrocycle, which is prone to adsorb onto the surface of the substrate. The selection of the appropriate disperser is key to this synthesis route. Liang, Wang, and their colleagues established a complete set of methodologies for this purpose, with *N*,*N*-dimethylformamide (DMF) being chosen as the powerful disperser.^48,49^ The microscopy images confirm the monomolecular dispersion and anchoring of metal–macrocycles on carbon nanotubes (Figure 4a). The obtained electrocatalysts demonstrated superior performance for electrocatalytic CO_2_ reduction. Besides directly anchoring the metal–macrocycle, appending another conjugated π-system group (fused-ring groups in most cases) as the substitution of metal–macrocycle is another strategy. Robert and co-workers appended the pyrene group onto iron–porphyrin units through a short linker, where the conjugated pyrene confers π–π interaction with the carbon surface, and therefore indirectly immobilized iron–porphyrin sites to the conductive substrate (Figure 4b).^50^ It is important to note that π–π interactions occur between each metal–macrocycle. Consequently, when integrating the system, the concentration of the metal–macrocycle and the types of disperser must be carefully considered to prevent interference. Additionally, considering that the supramolecular π–π interaction is relatively weaker than covalent bonds, the stability issue of loading metal–macrocycles via π–π interaction deserves more attention.

**Figure 4:**
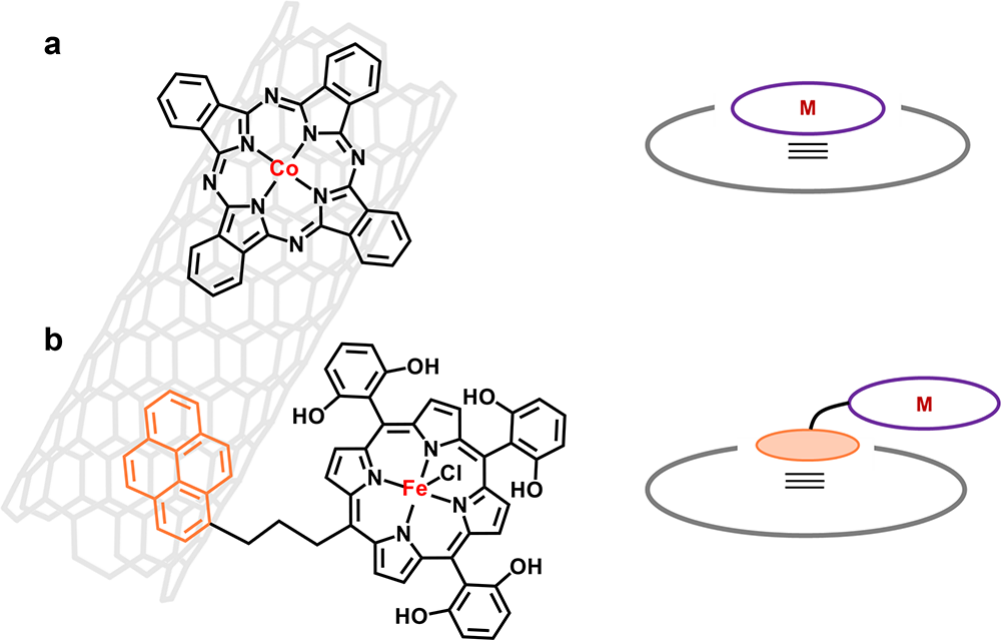
Utilizing π–π interactions to integrate the metal–macrocycle onto a carbon-based conductive substrate, regarding (a) directly loading the metal–macrocycle and (b) appending conjugated π-system substitution groups to indirectly integrate the metal–macrocycle.

Covalent immobilization is a systematic approach to immobilize metal–macrocycle units in a specific position. This method is advantageous due to its directional and saturated covalent bonds, which repel other metal–macrocycle units and thus promote monomolecular dispersion of the metal–macrocycle units and the construction of well-defined M–N–C sites. The core of covalent immobilization is the selection of a suitable splicing reaction to “weld” the metal–macrocycle onto the conductive substrate; an example of this was demonstrated by Li et al., who employed azide–alkyne cycloaddition reactions to successfully splice cobalt–corrole onto functionalized carbon nanotubes (Figure 5a).^51^ The obtained catalyst shows satisfactory activity for OER and hydrogen evolution (HER) electrocatalysis. Compared with azide–alkyne cycloaddition, the amidation reaction is a more easily performed splicing reaction, as the reaction substrate groups (carboxyl and the amino groups) are facile to functionalize on both carbon nanomaterials and metal–macrocycles.^52^ Maurin et al. covalently immobilized iron–porphyrin onto carbon nanotubes via amidation reaction and the obtained electrocatalyst facilitated electroreduction of CO_2_ into CO in neutral media (Figure 5b).^53^ However, the covalent immobilization linkage, in most cases, is a nonconjugated group and poor in electron conduction. Therefore, the length and type of the covalent immobilization linkage should be well-controlled to address such a drawback.

**Figure 5:**
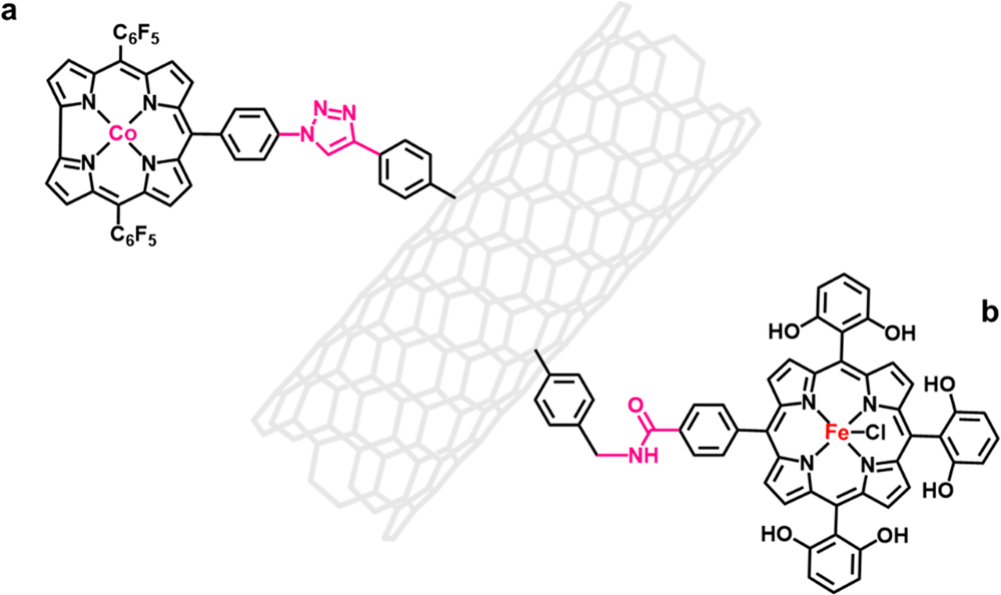
Covalent immobilization methodology to integrate M–N–C active sites, where different “splicing reactions” are selected, including (a) azide–alkyne cycloaddition reaction and (b) amidation reaction.

Reticular chemistry offers a distinct strategy from the two previously mentioned, as metal–porphyrin and metal–phthalocyanine are tetra-symmetric and can be woven into periodic frameworks with infinite permutations under the guidance of reticular chemistry. This interconnectedness of metal–macrocycles stabilizes them against undesired dissolution, thus ensuring full exposure of evenly arranged M–N_4_ active sites. In our previous contributions, cobalt–porphyrin was covalently linked into organic frameworks, known as cobalt-coordinated framework porphyrin (POF-Co, Figure 6a).^54^ Framework porphyrin is an intrinsically two-dimensional conjugated material and shows impressive compatibility with carbon-based materials due to its strong intermolecular π–π interactions. As a result, it can be in situ hybridized onto diverse carbon nanomaterials, functioning as the electrocatalysts^55^ or monolithic flexible electrodes.^56^ Furthermore, framework porphyrin can serve as an exquisite platform to compare the intrinsic electrocatalytic activity among diverse center metal atoms.^57^ Xiang and co-workers also fabricated a fully conjugated covalent organic framework with the infinite permutation of iron–phthalocyanine units (Figure 6b).^58^ The authors further composited the two-dimensional framework with graphene via their intermolecular interactions. The obtained electrocatalyst outperforms a platinum-based electrocatalyst for ORR, demonstrating the feasibility of a pyrolysis-free M–N–C electrocatalyst for practical applications.

**Figure 6:**
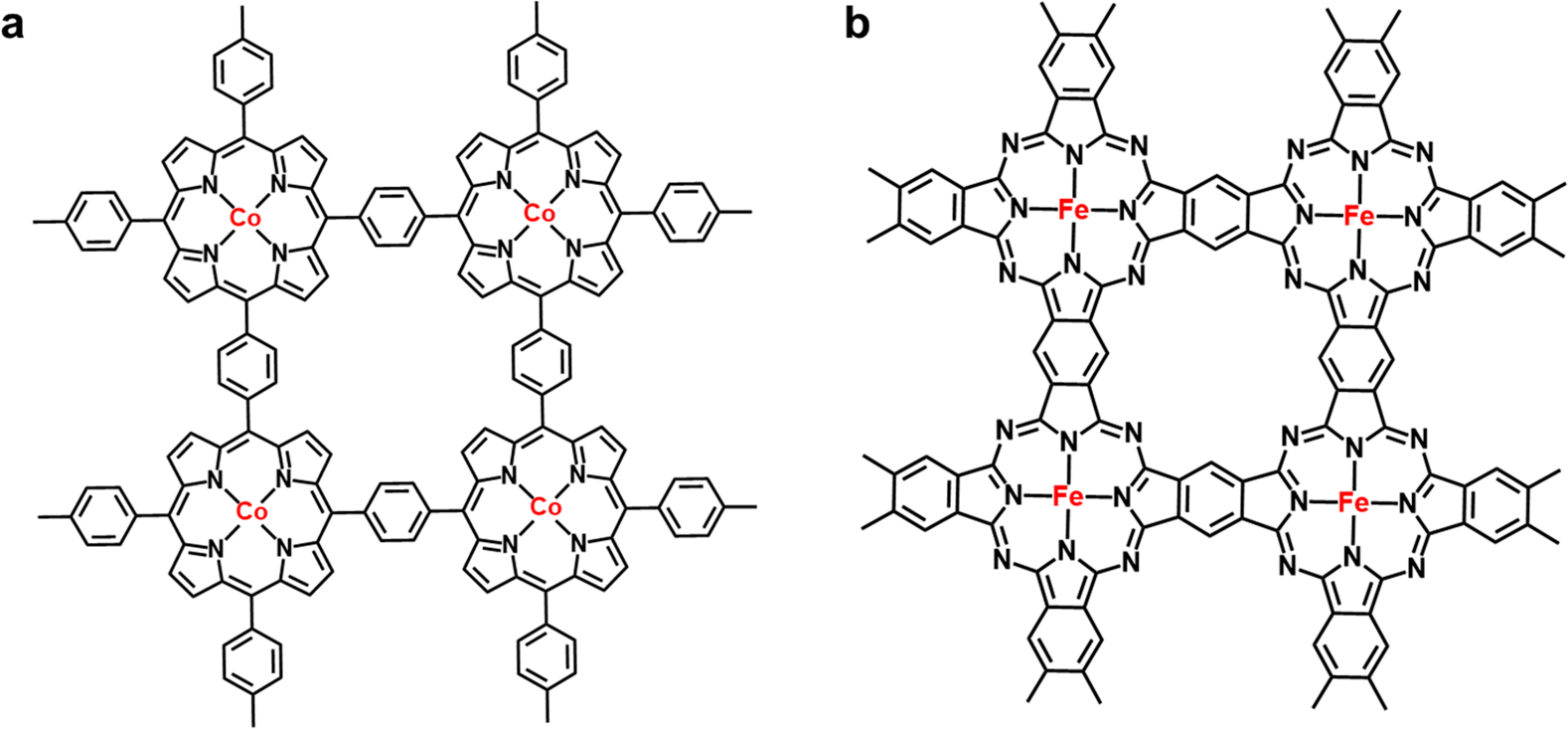
Reticular chemistry strategy to integrate metal–macrocycles into frameworks, e.g., (a) a cobalt-coordinated framework porphyrin and (b) an iron–phthalocyanine-rich covalent organic framework.

## 5 Conclusions and Outlook

For M–N–C catalysts as the next-generation electrocatalysts, their chemical synthesis without pyrolysis represents the highest level of precision achievable. Unlike the simple top-down pyrolysis process, the bottom-up chemical synthesis to craft the active sites offers great opportunities for molecular design, but it also presents huge challenges for practical applications. The current evidence provides a preliminary demonstration of the inherent advantages of structural precision and activity regulation, yet the application prospects of these catalysts remain open to question. Facing both opportunities and challenges, the ideal–reality gaps must be filled in through sustained effort. As such, the following recommendations are proposed.

(1) **Fundamental investigation.** Admittedly, the practical performances of pyrolysis-free M–N–C catalysts are not as good as those with pyrolysis in the current stages. However, the unique advantage of their well-defined and identical sites is irreplaceable, making them key to fundamental investigation on M–N–C catalysts and electrocatalysis. For instance, metal–macrocycles can be used as model samples to unambiguously figure out the direction of electronic structure regulation of M–N_4_ sites (e.g., electron donating or withdrawing). Moreover, the well-designed configuration of the sites can be used as a probe to detect the electrocatalytic mechanism by correlating the sites’ configuration and electrochemical performance. The hangman metal–macrocycle, for example, can decouple the electron transfer and proton delivery, thus simplifying the investigation into PCET processes. Utilizing metal–macrocycles as the research object, the structure–performance relationship can be revealed from the perspective of the semiconducting character.^59^ Efficient coordination regulation and dual-atom site construction can also be achieved based on heteroporphyrins and expanded porphyrins. We believe that pyrolysis-free M–N–C catalysts will bring more surprises and help us gain deeper insights into electrocatalysis.
(2) **Practical applications.** The alluring feature of pyrolysis-free M–N–C catalysts is that they offer limitless potential for attaining maximum and ultimate activity. To accomplish this, several issues must be addressed. First, the cause of the shortened activity is uncertain: is it the rapid loss of active sites, the limited site density, or unfavorable conductivity? Strategies to address this issue must be implemented urgently. Second, their poor stability is a persistent issue that necessitates extensive research, such as integrating metal–macrocycle sites into a monolithic electrode and ensuring its durability against high current densities. Finally, the molecular design and site integration of the metal–macrocycle are currently two distinct research fields. However, from a practical perspective, combining these two fields is necessary to obtain applicable electrocatalysts with both outstanding activity and stability. The rapid progress of precision chemistry is enabling pyrolysis-free M–N–C catalysts to achieve superior practical performance.
(3) **Bridging the gap.** The overemphasis on the differences between pyrolysis and pyrolysis-free M–N–C catalysts has led to a separation of research between the two. In reality, there is no unbridgeable gap between these two types of catalysts. Their respective research paradigms can provide valuable guidance for the investigation of the other. To bridge the gap, it is recommended that a more in-depth investigation of the structural transformation of the metal–macrocycle at different temperatures be conducted. Additionally, the pyrolysis procedure can be optimized with a variety of parameters to maintain the preconstructed structure while also increasing the carbonization degree for enhanced stability. This could allow for predesigned structures to be preserved during the pyrolysis process and to function similarly to pyrolysis-free catalysts. Lastly, structural modulation can also be applied to pyrolysis-obtained M–N–C catalysts, which is expected to combine the advantages of both pyrolysis and pyrolysis-free M–N–C catalysts. With the rapid advancement of synthesis and catalysis, it is time to put an end to the decades-long battle between pyrolysis and pyrolysis-free technologies.
(4) **Multidisciplinary and interdisciplinary studies.** Crafting pyrolysis-free M–N–C catalysts necessitates knowledge and expertise from various disciplines. Organic chemistry provides a solid foundation for bottom-up molecular design and chemical synthesis, while inorganic chemistry offers a theoretical basis to rationalize the coordination structure regulation. Moreover, catalysis chemistry, as a key branch of physical chemistry, elucidates the structure–performance relationship for both practical applications and conceptual advances. Energy chemistry ensures the applications of these catalysts in diverse cutting-edge energy devices, such as rechargeable batteries and electro-refinery. In addition, the research framework and research paradigm for pyrolysis-free M–N–C catalysts are shaped by other interdisciplinary fields, including polymer chemistry, computational chemistry, electrochemistry, and synthetic chemistry. To sum up, pyrolysis-free M–N–C catalysts require interdisciplinary integration and cooperation to function as both a model of precision chemistry and a state-of-the-art technique of chemical engineering.

In summary, the half-century-long battle between pyrolysis and pyrolysis-free technical routes has seen the rapid development of M–N–C catalysts. These pyrolysis-free M–N–C catalysts, which are distinct for their inherent precision, have contributed to the molecular design for activity enhancement and inspired researchers’ vision for the future. In this Perspective, we review their development history, discuss the inspiring contributions of precisely crafting the molecular structure, and highlight current efforts to address their stability issue via active site integration. We also present an overview of the current status of pyrolysis-free M–N–C catalysts and outline future research directions. We believe that precisely crafting pyrolysis-free M–N–C catalysts through interdisciplinary efforts not only is a remarkable breakthrough in electrocatalysis but also foreshadows the coming era of precise molecular manipulation in our near future.
